# Human Herpesvirus Replication and Abnormal CD8+ T Cell Activation and Low CD4+ T Cell Counts in Antiretroviral-Suppressed HIV-Infected Patients

**DOI:** 10.1371/journal.pone.0005277

**Published:** 2009-04-17

**Authors:** Mark A. Jacobson, Dirk P. Ditmer, Elizabeth Sinclair, Jeffrey N. Martin, Steven G. Deeks, Peter Hunt, Edward S. Mocarski, Caroline Shiboski

**Affiliations:** 1 Positive Health Program, Department of Medicine, University of California San Francisco and The Medical Service, San Francisco General Hospital, San Francisco, California, United States of America; 2 Department of Microbiology and Immunology, Lineberger Comprehensive Cancer Center and Center for AIDS research, University of North Carolina at Chapel Hill, Chapel Hill, North Carolina, United States of America; 3 Division of Experimental Medicine, Department of Medicine, University of California San Francisco, San Francisco, California, United States of America; 4 Department of Epidemiology and Biostatistics, University of California San Francisco, San Francisco, California, United States of America; 5 Department of Microbiology & Immunology, Emory University School of Medicine, Atlanta, Georgia, United States of America; 6 Department of Orofacial Sciences, School of Dentistry, University of California San Francisco, San Francisco, California, United States of America; New York University School of Medicine, United States of America

## Abstract

**Background:**

Most HIV-infected patients receiving virologically suppressive antiretroviral therapy continue to have abnormal, generalized T cell activation. We explored whether the degree of ongoing cytomegalovirus (CMV), Epstein-Barr virus (EBV) and Kaposi's sarcoma herpesvirus (KSHV) replication was associated with higher virus-specific T cell activation and the failure to achieve normal absolute CD4+ T cell counts in the face of long-term suppressive antiretroviral therapy.

**Methodology:**

Longitudinally collected PBMC and saliva specimens obtained from HIV-infected patients on effective antiretroviral therapy for at least one year (plasma HIV RNA <75 copies/mL) were examined using a multiplex CMV, EBV and KSHV DNA PCR assay. Eleven cases were chosen who had CD8+ T cell CD38+HLA-DR+ expression >10% and plateau absolute CD4+ T cell counts <500 cells/µL. Five controls from the same study had CD8+ T cell CD38 expression <10% and plateau absolute CD4+ T cell counts >500 cells/µL.

**Results and Conclusions:**

Among all subjects combined, 18% of PMBC samples were positive for CMV DNA, and 27%, 73% and 24% of saliva samples were positive for CMV, EBV and KSHV DNA, respectively. No significant differences or trends were observed between cases and controls in proportions of all CMV, EBV or KSHV DNA positive specimens, proportions of subjects in each group that intermittently or continuously shed CMV, EBV or KSHV DNA in saliva, or the median number of genome copies of CMV, EBV and KSHV DNA in saliva. Overall, number of genome copies in saliva were lower for KSHV than for CMV and lower for CMV than for EBV. Although replication of CMV, EBV and KSHV persists in many antiretroviral-suppressed, HIV-infected patients, we observed no evidence in this pilot case-control study that the magnitude of such human herpesvirus replication is associated with abnormally increased CD8+ T cell activation and sub-normal plateau absolute CD4+ T cell counts following virologically suppressive antiretroviral therapy.

## Introduction

Currently available highly active antiretroviral therapy (HAART) regimens are able to dramatically decrease plasma HIV RNA levels in the majority of patients with chronic HIV infection, leading to sustained increases in absolute CD4+ T cell counts and decreased risk of progression to AIDS and death. These antiretroviral regimens, however, have limitations in terms of immunologic efficacy [1]. Most patients whose absolute CD4+ T cell counts are restored to normal range continue to have elevated levels of T cell activation (as evidenced by the proportion of T cells expressing CD38 and HLA-DR) and abnormally elevated levels of B cell activation (as evidenced by elevated serum IgG concentrations) [2], [3]. This persistent immune activation during therapy likely has clinical significance, as it does in untreated HIV infection [4], [5]. For example, the degree of this persistent T cell activation is inversely correlated with the extent of absolute CD4+ T cell count reconstitution [2]. The clinical sequelae of chronic T and B cell activation in HAART-suppressed patients remain undefined, but soluble markers of immune activation have been associated with an increased risk of death and cardiovascular disease in this setting [6], and chronic activation has also been associated with an increased risk of autoimmune disease and cancer in HIV-uninfected populations.

The reasons underlying persistent T and B cell activation, in the face of effective antiretroviral therapy, are unknown. Potential causes include 1) persistent HIV replication, at levels below those detectable using currently available commercial plasma HIV RNA assays, 2) persistent systemic replication of a co-infecting virus such as human herpes or hepatitis viruses, 3) persistent microbial translocation [7], and/or 4) persistent B and T cell dysregulation that is antigen-independent. Since CMV-specific CD8 T-cell responses (as measured by the proportion of cells expressing IFNγ after stimulation with pp65 peptides) are higher in HAART-treated HIV-infected subjects compared to matched uninfected controls [8] and since CMV-specific CD8+ T cell activation and differentiation appear elevated during HIV infection [9], we investigated whether evidence of CMV replication in blood or saliva might be associated with the degree of peripheral blood CD8+ T cell activation and absolute CD4+ T cell count recovery in HIV-infected patients whose plasma HIV RNA had been suppressed by HAART below the level of detection for at least one year.

We chose saliva as a compartment to monitor active CMV replication because of the expectation that this body fluid would provide a sensitive source of this virus in antiretroviral-suppressed HIV-infected patients [10], [11] as it is in many other settings. We evaluated CMV DNA levels associated with PBMC instead of plasma because of the increased sensitivity afforded by this compartment [12], [13].

Finally, given the fact that both EBV or KSHV are also shed in increased amounts in saliva in HIV-infected patients [9], [11] and that EBV-specific CD8+ T cell activation increases in HIV infected patients [9], [14], we also assessed the DNA levels of these human herpesviruses in the same saliva samples.

## Methods

### Subjects

This study was conducted according to the principles expressed in the Declaration of Helsinki. The study was approved by the Institutional Review Board of the University of California San Francisco. All patients provided written informed consent for the collection of samples and subsequent analysis.

PMBC and saliva specimens were obtained from a prospective observational study of HAART (SCOPE). In SCOPE, subjects are evaluated by medical history and measurement of plasma HIV viral load and absolute CD4+ T cell count every 4 months. At each of these visits, samples of blood for plasma and PBMC separation and of unstimulated whole saliva are obtained and stored at −80°C. For this study we identified specimens from SCOPE participants who met the following criteria:

1. On HAART with at least one year of sequential plasma HIV RNA levels consistently sustained at levels <75 copies/mL before the time the first specimens tested were obtained.
2. Paired stored PBMC and saliva specimens were available from at least 2 separate visits spanning a minimum 6 month period while the subject remained on HAART with a HIV RNA level consistently <75 copies/mL and during which the percentage of circulating CD8+ T cells co-expressing CD38 and HLA-DR was measured.

Among the specimens from these participants, we selected “cases” who had high CD8+ T cell activation and low absolute CD4+ T cell counts at the time specimens tested were obtained, and were defined as:

- Percentage of circulating CD8+ T cells co-expressing CD38 and HLA-DR was >10%.
- Absolute CD4+ T cell counts were consistently <500 cells/µL.
- In addition, since chronic hepatitis C infection has been reported to cause abnormally increased CD8+ T cell activation and low absolute CD4+ T cell count in HIV-coinfected patients [2], only subjects who tested negative for hepatitis C antibody were included in Group 1.

We also identified a group of controls who had low CD8+ T cell activation and high absolute CD4+ T cell counts at the time specimens tested were obtained, defined as:

- Percentage of circulating CD8+ T co-expressing CD38 and HLA-DR was <10%
- Absolute CD4+ T cell counts were consistently >500 cells/µL.

### Immunophenotyping of Activated T cells

Freshly drawn EDTA anti-coagulated whole blood was stained, lysed and fixed as previously described [2]. Activated (CD38+HLA-DR+) CD8+ T lymphocytes were analyzed by 4-color flow cytometry on a Beckman Coulter Epics XL flow cytometer, using FITC-conjugated anti-HLA-DR, PE-conjugated anti-CD38 (both BD Bioscience), CY5-conjugated anti-CD3, and ECD-conjugated anti-CD8 (both Beckman Coulter) monoclonal antibodies. Appropriate isotype controls were used to define the positive CD38 and HLA-DR populations.

### CMV DNA PCR Assay of PBMC

As previously described [15], we amplified viral DNA from clinical samples with a low background and a sensitivity of 3 to 5 CMV genomes/10^5^ cell equivalents. Aliquots of 10^6^ freshly separated PBMC were lysed, boiled for 5 min, cooled on ice, and stored at −80°C until use. DNA was isolated from samples using the QIAGEN DNeasy Tissue kit (QIAGEN Inc., Valencia, CA) according to the manufacturer's instructions. The initial amplification of DNA was with primers IEP2AII (5′ATGGAGTCCTCTGCCAAGAGAAAGATGGAC3′) and IEP4BII (5′CAATACACTTCATCTCCTCGAAAGG3′), followed by IEP3B (5′TCTGCCAGGACATCTTTCTC3′ and IEP3A (5′GTGACCAAGGCCACGACGTT3′). For the initial qualPCR, 5 µl (1 µg; 10^5^ cell equivalents) of extracted PBMC and PMN DNA or 50 ng EMB DNA was added to 45 µl reaction mixtures containing 10 mM Tris-HCl (pH 8.3), 50 mM KCl, 1.5 mM MgCl2 (Roche Diagnostics Corporation, Indianapolis, IN), 200 µM of each deoxynucleotide triphosphate (Invitrogen Corporation), 1 U *Taq* polymerase (Roche Diagnostics Corporation), and 1 µM of each primer. For the second qualPCR, 5 µl out of the first reaction mixture was added to a 45 µl reaction mixture as used for the initial qualPCR. qualPCR mixtures were layered with 50 µl of mineral oil and subjected to one cycle of 94°C for 3 min, followed by 30 cycles of 1 min of denaturation at 94°C, 1 min of annealing at 62°C, and 2 min of extension at 72°C, and a final extension step of 7 min at 72°C. Positive and negative controls were included in each run, and, after qualPCR, each sample was electrophoresed through a 2% agarose gel containing 0.2 µg/ml ethidium bromide with appropriate DNA size markers. Amplicons were visualized on a UV transilluminator and photographed.

To quantify viral load in the qual-PCR-positive samples, we used SYBR green detection of real-time PCR products able to detect a range of 3 to 100,000 CMV genome copies/10^5^ cells. Real-time PCR was performed in a GeneAmp 5700 Sequence Detection System (Applied Biosystems, Foster City, CA). Five µl of sample was added to 45 µl of reaction mix containing 25 µl SYBR green PCR Master Mix (Applied Biosystems) and 10 pmol of each primer. The primers used for the real-time PCR were also located in the gene encoding the IE1 protein, exon 2 (IE1-2F, 5′GGCCGAAGAATCCCTCAAA3′; and IE1-2R, 5′TCGTTGCAATCCTCGGTCA3′). Conditions for the real-time PCR were established empirically and included incubation at 50°C for 2 min to enable uracil-DNA-glycosylase (in Master Mix) to act on samples and 94°C for 10 min to activate AmpliTaq Gold polymerase, followed by 40 cycles of 15 s of denaturation at 94°C and 1 min of annealing and extension at 60°C. A standard curve of serial dilutions of known amounts of plasmid DNA was used in each run as an internal control and to determine the copy number in the samples. In addition, a natural CMV DNA positive sample was run to assess consistency. The detection limit of this test was 5 CMV DNA copies/10^5^ cells, and positive samples were quantitated as copies/5 µL of lysed cells. Each run included positive and negative samples, and specificity of the amplified products was assessed on each run by dissociation curve analysis to ensure that all products had an expected, uniform melting temperature.

### Human Herpesvirus DNA PCR Assay of Saliva

DNA was isolated from 250 µl saliva (diluted to 1000 µl with PBS) using the QIAGEN QIAamp UltraSens Virus Kit (QIAGEN Inc., Valencia, CA) according to the manufacturer's instructions. DNA was eluted in 80 µl 0.1× TE pH 7.8 (Fisher Inc.). A single QPCR reaction contained 14 µl 2×SYBR (Roche Inc.), 1 µl primer (to yield 167 nM final concentration), 7 µl PCR grade water and 8 µl sample. Single plex reactions were set up on a RoboGo (MWG Inc.) pipetting station and analyzed on a MJR Opticon II machine. Cycle conditions were 56°C: 5 min, 95°C: 3 min, (95°C: 5 sec, 62°C: 30 sec, 40×). The primers were for EBV: EBNA3cF 5′- AAGGTGCATTTACCCCACTG, EBNA3cR, 5′- AGCAGTAGCTTGGGAACACC [16] for KSHV LANA78f 5′- GGAAGAGCCCATAATCTTGC, LANA78r 5′- GCCTCATACGAACTCCAGGT, FOR HCMV GlyBcmvF 5′-AAGTACCCCTATCGCGTGTG, GlyBcmvR 5′-ATGATGCCCTGATCCAAGTC [17]. A standard curve of serial dilutions of known amounts of target oligonucleotides was used to determine the copy number in the samples. Triplicates assays were performed with each saliva sample, and the median value was used in data analysis. Samples with melting temperature <76°C were excluded from the analysis. Samples with <1.8 log copies per ml were considered negative.

### Statistical Analysis

For analyses including multiple observations per participant, we evaluated differences in categorical outcomes (i.e., DNA detectable vs. undetectable) between cases and controls with logistic regression for clustered data and differences in DNA copy number between cases and controls with generalized estimating equations. For analyses comparing DNA copy numbers between groups, undetectable levels were assigned a value of 1.79 log_10_ copies/ml (just below the lower limit of detection of 1.8 log_10_ copies/ml). Fisher's exact tests were used to compare the proportion of cases and controls with ≥1, ≥2, or all samples positive. The Spearman rank correlation test was used to test the association of subject's percent of activated CD8+ T cells with the amount of herpesvirus DNA in their saliva.

## Results

Eleven SCOPE subjects meeting the criteria for inclusion were sampled as cases in the high CD8 T cell activation/low CD4 count group. The cases had 2–4 saliva and PMBC specimens assayed which spanned a 6–12 month period. The cases had a median (range) absolute CD4+ T cell count of 279 (112–450) cells/µL and percentage of activated (CD38+HLA-DR+) CD8+ T cells of 17.9% (11–38%) during this study period. Five subjects met the criteria for inclusion as controls in the lower CD8 T cell activation/higher CD4 count group, had 4 PMBC and saliva specimens assayed which spanned a 12 month period, and had a median (range) absolute CD4+ T cell count of 687 (519–858) cells/µL and percentage of activated (CD38+HLA-DR+) CD8+ T cells of 5.4% (4–7.6%) during this study period. Demographics and characteristics of the subjects studied are summarized in Table 1. Of note, there was a trend toward longer duration of sustained undetectable plasma HIV RNA on antiretroviral therapy in the control group (58 vs. 31 months, p = 0.06).

**Table 1:** Subject Demographics and Characteristics at time of first specimen.

	High CD8+/CD38+%-	Low CD8+/CD38+%-
	Low Abs. CD4+ count	High Abs. CD4+ count
N	11	5
Age (median, range years)	49 (42–62)	48 (39–54)
Male∶Female	10∶1	5∶0
White∶Black∶Hispanic∶Asian/Pacific Islander	8∶2∶1∶0	2∶1∶0∶2
Absolute CD4+ T cells/µL (median, range)	279 (112–450)	687 (519–858)
Percent of CD8+ T cells that are CD38+HLA-DR+ (median, range)	17.9 (11–38)	5.4 (4.0–7.6)
Months plasma HIV VL undetectable (median, range)	31 (16–53)	58 (24–74)

### Human Herpesvirus DNA PCR

Table 2 summarizes comparisons of the proportion of all case and control saliva and PBMC specimens that were positive (above the cut-off value of 1.8 log/mL of saliva for each of the three human herpesvirus DNA or ≥5 CMV DNA copies/10^5^ cells in PBMC), as well as comparisons of the proportion of case and control subjects who had ≥1, ≥2 or all saliva specimens positive. In addition, salivary herpesvirus DNA levels (log_10_ copies/mL) were compared between cases and controls using generalized estimating equations.

**Table 2:** Comparison of saliva and PBMC human herpes virus DNA PCR results between groups of HIV-infected, antiretroviral suppressed patients with high CD8+/CD38+% and low absolute CD4+ T cell counts versus those with low CD8+/CD38+% and high absolute CD4+ T cell counts.

	High CD8+/CD38+%-	Low CD8+/CD38+%-	P value
	Low Abs. CD4+ count	High Abs. CD4+ count	
**CMV DNA PCR**			
Positive saliva samples	10/35	5/20	0.77
Positive PBMC samples	8/40	3/20	0.61
Pts with ≥1 positive saliva	5/11	4/5	0.31
Pts with ≥2 positive saliva	4/11	1/5	1.00
Pts with all positive saliva	0/11	0/5	-
Mean within-subject mean saliva CMV DNA copy number (log10 copies/mL)	1.89	1.91	0.73
**EBV DNA PCR**			
Positive saliva samples	25/35	15/20	0.83
Pts with ≥1 positive saliva	10/11	5/5	1.00
Pts with ≥2 positive saliva	8/11	4/5	1.00
Pts with all positive saliva	5/11	2/5	0.55
Mean within-subject mean saliva EBV DNA copy number (log10 copies/mL)	3.16	3.38	0.74
**KSHV DNA PCR**			
Positive saliva samples	9/35	4/20	0.61
Pts with ≥1 positive saliva	7/11	3/5	1.00
Pts with ≥2 positive saliva	2/11	1/5	1.00
Pts with all positive saliva	0/11	0/5	-
Mean within-subject mean saliva KSHV DNA copy number (log10 copies/mL)	2.0	2.1	0.55

Among all subjects combined, 18% of PMBC samples were positive for CMV DNA and 27%, 73% and 24% of saliva samples were positive for CMV, EBV and KSHV DNA, respectively. However, all categorical comparisons between the case and control groups were non-significant with p values >0.5, and all comparisons of median DNA copy number between cases and controls were non-significant with p values >0.9.

As there was no consistent difference between the cases and controls in terms of any of the herpesviruses measured, we combined the groups to determine if the level of CMV, EBV or KSHV DNA in saliva was associated with the level of CD8+ T cell activation. The time point of saliva collection temporally closest to that of a CD8+ T cell CD38/HLA-DR expression measurement was used. We found no correlation between the percentage of activated CD8+ T cells and the amount of CMV DNA (r = 0.193, p = 0.47), EBV DNA (r = −0.254, p = 0.34), or KSHV DNA (r = 0.259, p = 0.33) in saliva.

In general, positive readings tended to occur within subjects, suggesting that the “steady-state” levels varied by subject. Of note, all positive PBMC samples had very low copy numbers, <3 CMV DNA copies per 5 µL of lysed cells. Also, the number of genome copies in saliva overall were lower for KSHV than for CMV and lower for CMV than for EBV.

## Discussion

Replication of CMV, EBV and KSHV persists in many antiretroviral-suppressed, HIV-infected patients to a greater degree than in HIV-uninfected adults [9]. T cell activation is also consistently higher in antiretroviral-suppressed patients compared to HIV negatives, and when present is often associated with suboptimal gains in absolute CD4+ T cells. To explore the possible causal role of persistent herpesvirus replication on immune activation and immune reconstitution during suppressive antiretroviral therapy, we selected two distinct groups of patients: those with relatively normal CD8+ T cell activation/high absolute CD4+ T cell counts and those with high CD8+ T cell activation/low absolute CD4+ T cell counts. Using very sensitive measures of herpesvirus persistence, we found no consistent association between the level of viral DNA and immune activation. We also found no evidence suggesting that residual viral replication during suppressive antiretroviral therapy was causally associated with either high T cell activation. The proportions of all CMV, EBV or KSHV DNA positive specimens between the cases and controls were essentially the same, as were the proportions of subjects in each group that intermittently or continuously shed CMV, EBV or KSHV DNA in saliva. In addition, the median number of copies of CMV, EBV and KSHV DNA longitudinally in cases was virtually identical to that in controls.

Miller, et al, previously reported results using a similar PCR assay to detect CMV, EBV and KSHV in similar unstimulated whole saliva specimens from 58 HIV-infected patients, most of whom had HIV replication suppressed by antiretroviral therapy [11]. In that study, 31%, 57% and 90% of saliva samples were positive for CMV, KSHV and EBV DNA, respectively, compared to 37%, 26% and 71% in our study. Perhaps, the lower number of positive KSHV and EBV specimens we observed was a result of our including only patients whose HIV replication had been completely suppressed below the limit of plasma detectability by commercial assay for at least one year.

Similar to the results from Miller et al, we noticed that median CMV levels were lower than median KSHV levels, which in turn were lower than median EBV levels (Table 2). This suggests that even though herpesvirus shedding is often considered a categorical event (i.e., a person either has undetectable viral load or is actively shedding virus), the amount of virus that can be transmitted orally by a person experiencing viral reactivation differs considerably for the different herpesviruses.

This pilot study has several important limitations. First and foremost, the sample size was small, and thus the potential Type II error is likely substantial. However, we avoided confounding factors by requiring prolonged HIV RNA undetectability in plasma for study eligibility and excluding patients with chronic HCV infection. Second, we selected our cases and controls based on more than one factor (i.e., the groups differed based on both CD8+ T cell activation and absolute CD4+ T cell counts). This study design would have been particularly problematic had we observed differences between the two groups. Since it is unlikely that low absolute CD4+ T cell counts may have blunted the impact of herpes viruses on immune activation, it is also unlikely that the negative results observed in this study were due to our study design. Third, we only measured human herpesvirus replication in two reservoirs, saliva and PBMC. Assaying specimens from other reservoirs (e.g., genital secretions, urine, plasma) may have demonstrated a difference between the two groups. In addition, the saliva and PBMC specimens we assayed were obtained infrequently (only once every four months). Sampling more frequently, for example by daily oral swabs [10], might reveal differences between these two groups of patients in the frequency and duration of herpesvirus replication. Since it is impractical to assay all potential reservoirs of human herpesvirus daily, an alternative method to determine the role of human herpesvirus replication in driving abnormal T cell activation in HIV-infected patients would be to administer therapy with an agent with broad anti-herpesvirus activity, such as valganciclovir, and then measure the effect on T cell activation and absolute CD4+ T cell count in a randomized, controlled manner. We are currently performing such a trial.

Given the strong association between inflammation/immune activation and disease outcomes in treated HIV disease, defining the mechanisms associated with HIV-associated inflammation is of high importance. Our preliminary data do not provide strong evidence for a role of persistent high level herpesvirus replication.

Other possible mechanisms includes the following:

1. Persistent microbial translocation. It is now well-appreciated that HIV causes irreversible damage to the gut mucosal integrity early in the course of disease, leading to translocation of gut microbial products into the system circulation [18]. Circulating lipopolysaccharide and bacterial DNA have each been reported to correlate with measures of T cell activation in HIV-infected patients [7], [19].
2. Residual HIV viremia. Another factor leading to persistent abnormal immune activation in antiretroviral-suppressed patients could be persistent low level replication of HIV. Several studies have reported that by using an ultra-ultrasensitive HIV RNA PCR assay, low levels of plasma HIV viremia (below the detectability of currently available commercial HIV RNA assays) can be detected in the majority of HIV-infected patients successfully treated with antiretroviral therapy [20]–[24]. Results of these studies indicate that the level of this residual viremia remains stable for at least up to seven years [23], [24]. Of note, the duration of suppressive antiretroviral therapy in the controls we studied tended to be longer than in the cases, which could be consistent with the controls having a lower level of HIV replication than the cases.
3. Persistant immune dysregulation. Finally, the damage to the immune system done by uncontrolled HIV replication before antiretroviral therapy is initiated, for example in altering T cell diversity in adults with limited thymic reserve for repopulating diverse naïve T cells [25] or reducing the number of regulatory T cells [26]–[29], could result in persistent T cell dysregulation that is antigen-independent.

In summary, future studies to examine the effect of suppressing CMV, EBV and KSHV with drugs such as valganciclovir [10] may better clarify the role of persistent human herpesvirus replication in driving abnormal T cell activation and resultant incomplete immune reconstitution in patients with HIV infection. Furthermore, observational studies that measure HIV RNA by ultra-ultrasensitive assays [20], circulating lipopolysaccharide and/or bacterial DNA, and measurements of regulatory T cells or T cell superfamily representation (a method to characterize T cell diversity) and correlate these parameters with measurements of T cell activation and absolute CD4+ T cell counts in cohorts of long-term, antiretroviral suppressed patients could help elucidate the etiologic contributions of microbial translocation, low level HIV replication, and persistent T cell dysregulation to this pathologic process.
